# Systems pathway engineering of *Corynebacterium crenatum* for improved L-arginine production

**DOI:** 10.1038/srep28629

**Published:** 2016-06-24

**Authors:** Zaiwei Man, Meijuan Xu, Zhiming Rao, Jing Guo, Taowei Yang, Xian Zhang, Zhenghong Xu

**Affiliations:** 1 The Key Laboratory of Industrial Biotechnology, Ministry of Education, School of Biotechnology, Jiangnan University, Wuxi 214122, China; 2 State Key Laboratory of Food Science and Technology, Jiangnan University, Wuxi 214122, China; 3 School of Pharmaceutical Sciences, Jiangnan University, Wuxi 214122, China

## Abstract

L-arginine is an important amino acid in food and pharmaceutical industries. Until now, the main production method of L-arginine in China is the highly polluting keratin acid hydrolysis. The industrial level L-arginine production by microbial fermentation has become an important task. In previous work, we obtained a new L-arginine producing *Corynebacterium crenatum* (subspecies of *Corynebacterium glutamicum*) through screening and mutation breeding. In this work, we performed systems pathway engineering of *C. crenatum* for improved L-arginine production, involving amplification of L-arginine biosynthetic pathway flux by removal of feedback inhibition and overexpression of arginine operon; optimization of NADPH supply by modulation of metabolic flux distribution between glycolysis and pentose phosphate pathway; increasing glucose consumption by strengthening the preexisting glucose transporter and exploitation of new glucose uptake system; channeling excess carbon flux from glycolysis into tricarboxylic acid cycle to alleviate the glucose overflow metabolism; redistribution of carbon flux at α-ketoglutarate metabolic node to channel more flux into L-arginine biosynthetic pathway; minimization of carbon and cofactor loss by attenuation of byproducts formation. The final strain could produce 87.3 g L^−1^ L-arginine with yield up to 0.431 g L-arginine g^−1^ glucose in fed-batch fermentation.

L-arginine, a semiessential amino acid, has numerous applications in food flavoring and pharmaceutical industries. In humans, L-arginine is a conditionally essential amino acid for protein synthesis, and its metabolism also gives rise to nitric oxide, a key component of endothelium-derived relaxing factor. Thus, L-arginine can be also utilized in many clinical areas such as relax and dilate blood vessels^1^,^2^.

L-arginine can be produced by starting from keratinous proteins in human hair, pig bristles or animal feathers via acid hydrolysis, like L-cysteine^3^. In China, due to the abundant sources of keratinous proteins and low manufacturing costs, L-arginine is nowadays mainly produced by the highly polluting keratin hydrolysis. However, environmental pollution is an urgent global problem now. With the constant improvement of the environmental protection consciousness and the further development of green and sustainable industrial biotechnology^4^, the industrial level L-arginine production by environmentally friendly and economic feasible biotechnology has become an important task^1^.

Like most of the other amino acids, L-arginine can also be produced by microbial fermentation, employing mutant strains of *Corynebacterium*^1^,^5^,^6^. To achieve the industrial level production of L-arginine based on microbial fermentation, the microbial processes must possess high product concentration, high yield and high productivity properties^7^.

L-arginine is biosynthesized from L-glutamate through ornithine and citrulline in cellular metabolic pathways (Fig. 1). The genes involved in L-arginine biosynthesis in *Corynebacterium glutamicum* are organized as a gene cluster *argCJBDFRGH* and divided into two separate parts, which are *argCJBDFR* and *argGH* operons. The gene cluster encodes all of the enzymes required to convert L-glutamate to L-arginine^6^,^8^. In recent years, due to the development of recombinant DNA techniques and increased understanding of the biochemistry of metabolic reactions, metabolic engineering has strongly contributed to the performance of *C. glutamicum* in industrial production, including modifications of terminal production pathways as well as flux redirection for elimination of undesired by-products or enhancing supply of building blocks, redox power or energy^1^,^6^,^9^. Many metabolic engineering efforts aiming to enhance L-arginine production have been carried out. For example, deletion of *argR* (encoding the repressor of L-arginine biosynthesis operon) and alleviation of feedback inhibition of N-acetylglutamate kinase (NAGK, encoded by *argB*) were performed in *C. glutamicum* ATCC 13032, the constructed strain could produce 52 g L^−1^ of L-arginine^10^. Plasmid-based overexpression of the *argB*_M3_ (encoding the feedback-resistant NAGK) in L-arginine producing *C. crenatum* SYPA 5-5 led to a 41.7% increase of L-arginine production, reaching 45.6 g L^−1 ^5. Plasmid-based overexpression of the *argCJBDFRGH* cluster in *C. crenatum* SYPA 5-5 also increased the L-arginine production^8^. A recent systems metabolic engineering of *C. glutamicum* involving removal of regulatory repressors of L-arginine operon, optimization of NADPH level, disruption of L-glutamate exporter and flux optimization of rate-limiting L-arginine biosynthetic reactions led to a very successful production of L-arginine, the final constructed strain produced 92.5 g L^−1^ of L-arginine with a yield of 0.40 g g^−1^ glucose and productivity of 1.28 g L^−1^ h^−1^ at the laboratory-scale fermentations, and these are the highest concentration, yield and productivity of L-arginine reported to date^1^,^6^.

The tricarboxylic acid (TCA) cycle produces α-ketoglutarate and oxaloacetate as precursors of the glutamate family and the aspartate family amino acids, respectively, and other important intermediates such as succinyl-CoA^11^. The TCA cycle has not been rationally engineered for L-arginine biosynthesis so far, despite its major role in *C. glutamicum*. However, the TCA cycle might offer a great potential for optimization. In fermentation process, the byproduct formation could not only decrease the production of desired product but also increase the carbon loss and the difficulties of downstream processing^12^. For targeted downregulation of byproduct formation pathway, carbon flux is mainly blocked by gene deletion. But the deletion of growth-required reactions from the carbon core metabolism can induce undesired side effects such as growth deficiency or extended nutrient requirement^13^. The promoter, ribosome binding site (RBS) and translational start codon of enzyme-coding genes directly affect the intracellular activity of the encoded enzyme, this could be exploited to increase or attenuate the enzyme activities in order to redirect the carbon flux from undesired or competing pathways toward reactions supporting the desired product formation^9^,^13^,^14^.

In our previous work, a new L-arginine producing *Corynebacterium crenatum* (subspecies of *C. glutamicum*) was isolated from soil sample, and after a series of mutation breeding the mutant strain SYPA5-5 could produce 30.6 g L^−1^ L-arginine under optimal fermentation conditions^5^,^15^. In SYPA5-5, the transcriptional repressor ARGR encoded by *argR* gene is inactive, because a nucleotide substitution (C→T) mutation in the *arg*R gene created an early termination codon compared to the wild type^16^.

In this study, we conducted pathway engineering on *C. glutamicum* SYPA5-5 for achieving the three important objectives in L-arginine production by fermentation: high product concentration, high yield and high productivity. Stepwise rational pathway engineering based on the analysis of cellular metabolism and major metabolites accumulation in L-arginine fermentation resulted in gradual increase in L-arginine production throughout the strain engineering steps. Batch fermentation of our final strain constructed in this study without optimization of fermentation medium and conditions, the L-arginine production could reach to 87.3 g L^−1^. Pilot-scale fermentation of the final strain resulted in 78.4 g L^−1^ of L-arginine with a productivity of 0.98 g L^−1^ h^−1^ and yield of 0.387 g g^−1^ glucose. Combine the cost analysis, the results showed that L-arginine production by fermentation of the final strain constructed in this paper has great potential to substitute the keratin hydrolysis method in China.

## Results and Discussion

### Optimization of L-arginine biosynthetic pathway

The N-acetylglutamate kinase (NAGK) encoded by the *argB* gene is feedback inhibited by L-arginine in *C. glutamicum*^5^,^6^. In our previous work, this feedback inhibition was removed by site-directed mutagenesis of NAGK, and L-arginine production was largely enhanced by plasmid-encoded overexpression of the multi-mutated NAGK_M3_^5^. Thus, we used the mutated *argB*_M3_ to replace the native *argB* in the *C. crenatum* SYPA5-5 (Cc0 strain), resulting strain CcMB. This strain showed enhanced L-arginine production and faster glucose consumption (Table S1).

As described above, the repression of the L-arginine biosynthesis operon by the regulator ARGR (encoded by *argR*) is removed in *C. crenatum* SYPA5-5, because the ARGR is inactive^16^ and the L-arginine production can be increased by plasmid-encoded overexpression of the arginine operon^8^. In order to amplify the L-arginine biosynthetic flux, the genes involved in L-arginine biosynthesis were overexpressed by replacing the native promoters of *argCJBDFR* and *argGH* operons with the promoter of *eftu*, encoding elongation factor tu^17^ in the CcMB strain to make the Cc1 strain. The expression of genes in *argCJBDFR* and *argGH* operons were strengthened in Cc1 strain at the transcriptional level by promoter replacement (Fig. 2A) and this resulted in enhanced L-arginine productivity (Table S2). Fed-batch fermentation of the Cc1 strain resulted in the production of 53.2 g L^−1^ L-arginine which is 29% greater than that produced by the Cc0 strain. In addition, the glucose consumption rate and the L-arginine yield on glucose were also increased (Fig. 3 and Table 1), and the byproducts formation simultaneously decreased (Table 2).

### Optimization of the metabolic flux distribution between glycolysis and pentose phosphate pathway

**Figure 1:**
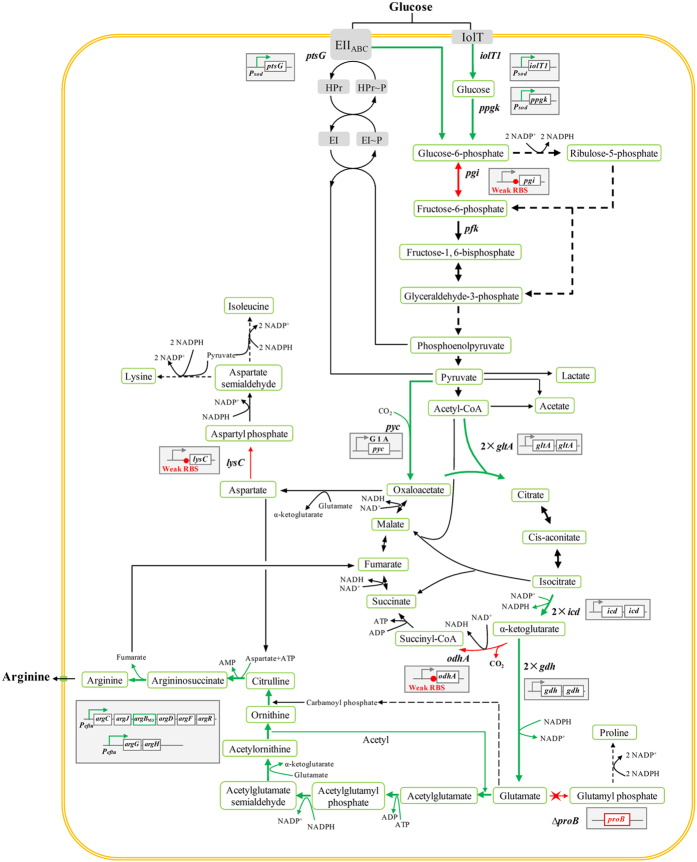
Schematic representation of the L-arginine biosynthesis pathway of *C. crenatum* and of the metabolic engineering steps performed in this study. Gray boxes represent the targeted modifications of the genes and all modifications were implemented into the genome. Green arrows indicate amplification, red arrows indicate attenuation or deletion, and the symbol “×” indicates gene deletion. Genes and enzymes: *argB*, N-acetylglutamate kinase; *pfk*, phosphofructokinase; *pgi*, phosphoglucoisomerase; *ptsG*, glucose-specific EIIABC^Glc^ component; *iolT1*, inositol permease; *ppgk*, glucose kinase; *pyc*, pyruvate carboxylase; *gltA*, citrate synthase; *icd*, isocitrate dehydrogenase; *gdh*, glutamate dehydrogenase; *odhA*, α-ketoglutarate dehydrogenase; *lysC*, aspartokinase; *proB*, glutamate kinase.

For efficient production of L-arginine, the supply of cofactor NADPH is one of the critical factors (Fig. 1)^6^. The NADPH is generated mainly through pentose phosphate pathway (PPP), and increasing the fluxes through PPP or redirection of carbon from glycolysis toward the PPP are effective to improve the intracellular NADPH regeneration and amino acid production^6^,^9^. However, The increased flux towards the PPP, also resulted in the inevitable loss of carbon in substrate.

The metabolic network is very complicated and can vary greatly in different strains^18^. To investigate whether the PPP flux in our strain is an limiting factor for NADPH regeneration and efficient L-arginine biosynthesis, we modulated the metabolic flux distribution between the glycolysis and PPP. Firstly, we constructed strain Cc1-2*pfk* for phosphofructokinase (PFK)^19^ overexpression to pull more flux from PPP into glycolysis. The PFK overexpression (Fig. 4A) resulted in increase of glucose consumption and cell growth, but decrease of L-arginine production, yield and intracellular NADPH level (Table S3). This result indicates that the adequate PPP flux is essential for high-efficiency L-arginine biosynthesis.

Then, we downregulated the expression level of the *pgi* gene encoding the first glycolysis-specific enzyme phosphoglucoisomerase (PGI)^20^ by ribosome binding site (RBS) substitution. In bacteria, RBS is one of the crucial elements for gene expression, it controls the translation initiation^14^. The theoretical strength predicted by RBS Calculator^14^ (https://www.denovodna.com/software/doLogin) of the natural RBS of *pgi* gene is 11290 au. Thus, the RBSs with strengths of 4000 au, 5000 au, 6500 au and 8500 au designed by the RBS Calculator were used to replace the natural RBS of *pgi* gene in Cc1 strain, resulting strains Cc2-4000, Cc2-5000, Cc2-6500 and Cc2-8500. The replacement of RBS had almost no effect on gene expression at the transcriptional level (Fig. 5A). As shown in Table S4, the specific PGI activities decreased with the attenuation of RBS strength, and the cell growth and glucose consumption decreased with the reduction of PGI activity, and the NADPH level increased gradually. The strain Cc2-6500 showed the highest L-arginine production, and the L-arginine yields of Cc2-4000, Cc2-5000 and Cc2-6500 strains were very similar to each other. This indicates that the flux ratio of PPP in the Cc2-6500 strain was sufficient for NADPH regeneration and L-arginine biosynthesis. Fed-batch fermentation of the Cc2-6500 strain allowed production of 66.4 g L^−1^ L-arginine with a yield of 0.325 g g^−1^ (Table 1). However, the cell growth and glucose consumption rate decreased, and the cultivation time was prolonged from 75 h to 96 h (Fig. 3). In addition, the formation of lysine, isoleucine and proline was strongly increased (Table 2). The biosynthesis of these three byproducts all require the cofactor NADPH^9^,^21^,^22^. Thus, it can be speculated that the high intracellular NADPH level functioned as a driving force for the biosynthesis pathways of lysine, isoleucine and proline, probably is the reason for the observed significant improvement in their productions^23^.

### Increasing the glucose consumption

**Figure 2:**
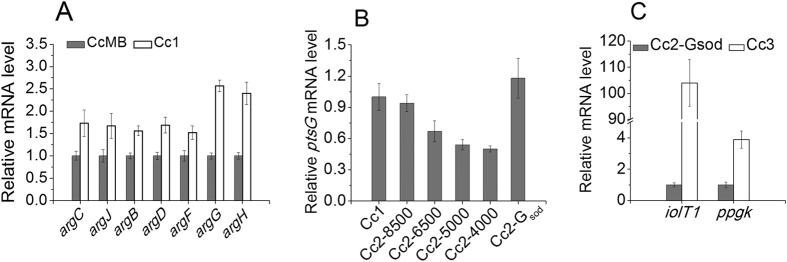
The effects of promoter replacement on the genes transcriptional level. (**A**) Comparison of transcriptional levels of L-arginine biosynthesis genes in CcMB and Cc1 strains. (**B**) Comparison of *ptsG* transcriptional levels in Cc1, Cc2 and Cc2-G_sod_ strains. (**C**) Comparison of *iolT1* and *ppgk* transcriptional levels in Cc2-G_sod_ and Cc3 strains. The transcriptional levels of genes were determined by RT-PCR, and presented as relative normalized expression. Error bars based on three biologically independent experiments.

The slow glucose consumption of the Cc2-6500 strain results in prolonged fermentation time and a decrease in L-arginine productivity compared to the Cc1 strain. Lindner *et al*. reported that the phosphotransferase system (PTS)-mediated glucose uptake and *ptsG* (encoding glucose-specific EIIABC^Glc^ component) transcription are drastically repressed in PGI-deficient *C. glutamicum* strains, and the glucose uptake and cell growth can be restored by plasmid-encoded overexpression of *ptsG*^20^. Figure 2B shows that the transcription level of *ptsG* decreased with the downregulation of the *pgi* expression in Cc2 strains. In order to facilitate the *ptsG* expression in the Cc2-6500 strain, the native promoter of *ptsG* gene was replaced by the strong *sod* promoter^6^, resulting strain Cc2-G_sod_. This strain showed improved growth and glucose uptake (Table S5), and the *ptsG* transcription level was increased by promoter replacement (Fig. 2B).

**Figure 3:**
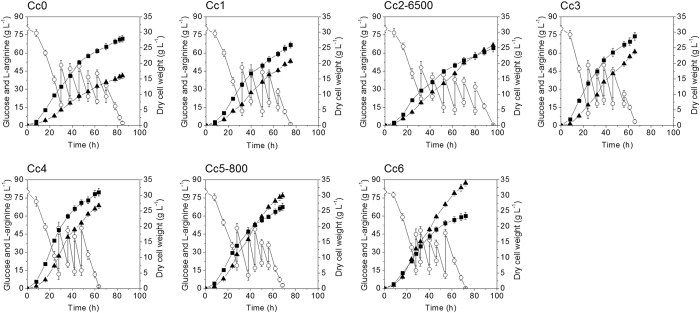
The L-arginine fed-batch fermentations of various *C. crenatum* strains. Signal denotes: L-arginine (filled triangles), dry cell weight (filled squares), glucose (open circles). Error bars based on three biologically independent experiments.

A different glucose uptake system that functions as an alternative to the PTS was also described in *C. glutamicum*. In this system, glucose is imported by two inositol permeases (IolT1 and IolT2), and phosphorylated via two glucose kinases Glk and PpgK, and plasmid-encoded overexpression of *ppgk* gene with either *iolT1* or *iolT2* gene in a PTS-deficient strain sustained fast growth in glucose^24^. The expression of IolT1 is repressed by the repressor IolR^25^. In order to further improve glucose uptake and L-arginine productivity, the native promoters of *iolT1* and *ppgk* gene were replaced by the *sod* promoter in the Cc2-G_sod_ strain, resulting strain Cc3. Figure 2C showed that the *iolT1* and *ppgk* transcription levels in Cc3 were improved by promoter replacement. This strain showed faster glucose consumption rate and higher productivity (Table S6). Fed-batch fermentation of the Cc3 strain allowed production of 61.0 g L^−1^ L-arginine with a productivity of 0.93 g L^−1^ h^−1^ and yield of 0.294 g g^−1^ (Fig. 3 and Table 1). Meanwhile, the formation of acetate and lactate increased (Table 2), the result indicates that the glucose overflow metabolism was provoked by the increased glucose uptake rate and glycolytic fluxes with a limited TCA cycle capacity^26^,^27^.

### Channeling carbon flux into TCA cycle

The glucose overflow metabolism leads to carbon wasting and hinders L-arginine synthesis. To alleviate the glucose overflow metabolism, the flux from glycolysis need to be channeled into the TCA cycle. Pyruvate carboxylase (PYC) encoded by *pyc*, is an important anaplerotic enzyme that catalyzes the carboxylation of pyruvate to form oxaloacetate (OAA). Overexpression of PYC can increase the accumulations of TCA cycle metabolite intermediates that can strengthen the TCA cycle and facilitate cell growth^28^. In addition, aspartate derived from OAA is required for L-arginine synthesis (Fig. 1). In order to channel more glycolytic flux into the TCA cycle, the PYC was overexpressed by substitution of the *pyc* native start codon GTG by ATG in Cc3, resulting strain Cc3-*pyc*_G1A_. The L-arginine production, cell growth and glucose consumption of Cc3-*pyc*_G1A_ was further increased, although the L-arginine yield on glucose decreased slightly (Table S7).

**Figure 4:**
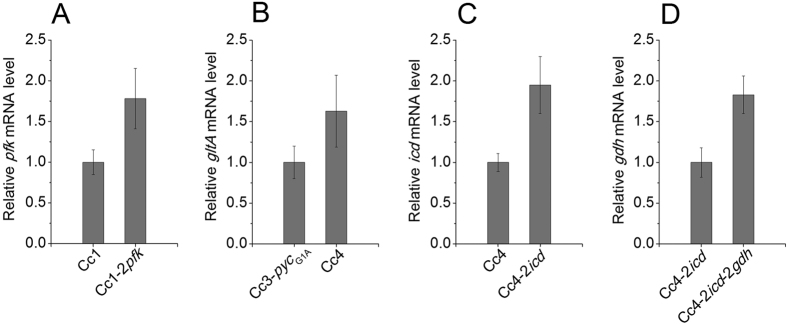
The effects of amplification of gene copy on the genes transcriptional level. (**A**) Comparison of *pfk* transcriptional levels in Cc1 and Cc1-2*pfk* strains. (**B**) Comparison of *gltA* transcriptional levels in Cc3-*pyc*_G1A_ and Cc4 strains. (**C**) Comparison of *icd* transcriptional levels in Cc4 and Cc4-2*icd* strains. (**D**) Comparison of *gdh* transcriptional levels in Cc4-2*icd* and Cc4-2*icd*-2*gdh* strains. The transcriptional levels of genes were determined by RT-PCR, and presented as relative normalized expression. Error bars based on three biologically independent experiments.

Citrate synthase (CS) encoded by *gltA*, catalyzes the initial reaction of the TCA cycle (Fig. 1), and it is considered to be rate controlling for the entry into the TCA cycle^29^. Overexpression of CS can redirect more carbon flux towards the TCA cycle in *C. glutamicum*^29^. In order to pull more carbon into the TCA cycle, in Cc3-*pyc*_G1A_ strain, the CS was overexpressed by implementation of an additional copy of *gltA* on chromosome, resulting strain Cc4 (Fig. 4B). This strain showed higher productivity and yield, and faster glucose consumption rate (Table S8). Fed-batch fermentation of the Cc4 strain allowed production of 68.6 g L^−1^ L-arginine with a productivity of 1.08 g L^−1^ h^−1^ and yield of 0.336 g g^−1^ (Fig. 3 and Table 1) and the accumulations of acetate and lactate were decreased significantly (Table 2). But, the formation of lysine and isoleucine was slightly increased, this might be because the overexpression of PYC increased the formation of aspartate, the precursor of lysine and isoleucine (Fig. 1).

### Carbon flux optimization of α-ketoglutarate metabolic node

α-ketoglutarate is a key intermediate in the TCA cycle, and occupies the branch point of the TCA cycle and L-arginine biosynthesis (Fig. 1). Therefore, the carbon flux distribution at the α-ketoglutarate metabolic node has a great potential for optimization to enhance the L-arginine biosynthesis. Isocitrate dehydrogenase (ICD) encoded by *icd*, oxidatively decarboxylates isocitrate to α-ketoglutarate and form an NADPH (Fig. 1), and it competes with the glyoxylate cycle enzyme isocitrate lyase for isocitrate^30^. In order to channel the isocitrate towards L-arginine synthesis, the ICD was overexpressed by implementation of an additional copy of *icd* in Cc4, resulting strain Cc4-2*icd* (Fig. 4C). This strain showed higher L-arginine production and yield, and faster glucose consumption rate, and the NADPH level was slightly increased (Table S9).

In *C*. *glutamicum*, glutamate dehydrogenase (GDH) encoded by *gdh*, catalyses the amination of α-ketoglutarate to L-glutamate. The extracellular glutamate production and intracellular glutamate concentration of *C*. *glutamicum* can be increased by overexpression of GDH^31^. In order to pull more α-ketoglutarate from TCA cycle into L-arginine biosynthesis, the GDH was overexpressed by implementation of an additional copy of *gdh* in Cc4-2*icd*, resulting strain Cc4-2*icd*-2*gdh* (Fig. 4D). Overexpression of GDH had a negative effect on cell growth, and the L-arginine production increased slightly. More α-ketoglutarate was used to form glutamate, the TCA cycle flux downstream of α-ketoglutarate and the formation of building blocks, redox power or energy for cell growth were decreased, and these may result in the slowdown of cell growth. However, the L-arginine production per gram of biomass and L-arginine yield on glucose increased obviously (Table S10).

**Figure 5:**
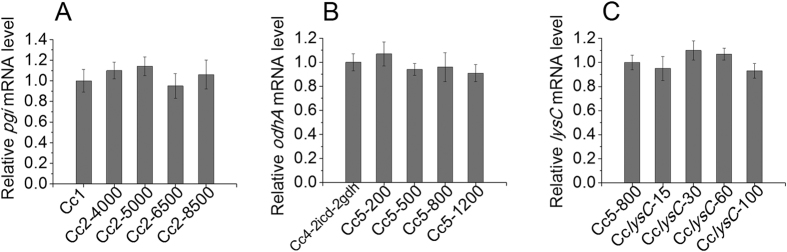
The effects of RBS replacement on the genes transcriptional level. (**A**) Comparison of *pgi* transcriptional levels in Cc1 and Cc2 strains. (**B**) Comparison of *odhA* transcriptional levels in Cc4-2*icd*-2*gdh* and Cc5 strains. (**C**) Comparison of *lysC* transcriptional levels in Cc5-800 and Cc*lysC* strains. The transcriptional levels of genes were determined by RT-PCR, and presented as relative normalized expression. Error bars based on three biologically independent experiments.

The α-ketoglutarate dehydrogenase complex (ODHC) catalyzes the oxidative decarboxylation of α-ketoglutarate to succinyl coenzyme A (succinyl-CoA), and *odhA* gene encodes the E1o subunit of the ODHC^31^. As stated above, channeling the carbon flux into L-arginine pathway by GDH overexpression at α-ketoglutarate metabolic node is beneficial for enhancement of L-arginine yield on glucose. Thus, in order to force more carbon flux towards L-arginine pathway, the ODHC activity needs to be attenuated. Similarly, attenuation of ODHC activity was carried out by optimization of *odhA* RBS. The theoretical strength of the natural RBS of *odhA* is 1613 au. Thus, the RBSs with strengths of 200 au, 500 au, 800 au and 1200 au designed by the RBS Calculator were used to replace the natural RBS of *odhA* in Cc4-2*icd*-2*gdh*, resulting strains Cc5-200, Cc5-500, Cc5-800 and Cc5-1200. The relative mRNA levels of *odhA* in Cc4-2*icd*-2*gdh* and Cc5 strains are shown in Fig. 5B. The specific ODHC activities, L-arginine production, biomass and L-arginine yield of the Cc4-2*icd*-2*gdh* and Cc5 strains are listed in Table S11. The results showed that it is effective to control the specific ODHC activity through the regulation of E1o subunit expression. And the cell growth and glucose consumption decreased with the reduction of ODHC activity. Among these strains, the strain Cc5-800 showed the highest L-arginine production. Fed-batch fermentation of the Cc5-800 strain allowed production of 76.8 g L^−1^ L-arginine with a productivity of 1.12 g L^−1^ h^−1^ and yield of 0.372 g g^−1^. Although the L-arginine productivity increased slightly, the L-arginine production per gram of biomass and L-arginine yield on glucose obviously increased (Fig. 3 and Table 1). Therefore, the overexpression of ICD and GDH and attenuation of ODHC activity can increase carbon flux into L-arginine pathway, and decrease the carbon flux into anabolism and carbon loss by CO_2_ release during oxidative decarboxylation of α-ketoglutarate.

### Minimization of carbon and cofactor loss

Lysine, isoleucine and proline are the main byproducts in L-arginine fermentation by Cc5-800 (Table 2). In *C. glutamicum*, lysine and isoleucine are synthesized from aspartate, and a large amount of NADPH is required for their synthesis. In addition, aspartate is also required for L-arginine synthesis (Fig. 1). Aspartokinase (AK) encoded by *lysC*, catalyzes the conversion of aspartate to L-aspartyl-phosphate, the first step in the biosynthesis of lysine and isoleucine^9^,^21^. The inactivation of AK has a strong negative effect on cell growth, because the lysine pathway intermediate diaminopimelate is an essential building block for the synthesis of cell wall^9^,^32^. Thus, attenuation rather than blocking of the lysine and isoleucine pathway flux at the level of AK has to be adopted. The theoretical strength of the natural RBS of *lysC* is 131 au. Thus, the RBSs with strengths of 15 au, 30 au, 60 au and 100 au designed by the RBS Calculator were used to replace the natural RBS of *lysC* in Cc5-800, resulting strains Cc5*lysC*-15, Cc5*lysC*-30, Cc5*lysC*-60 and Cc5*lysC*-100. The relative mRNA levels of *lysC* in Cc5-800 and Cc5*lysC* strains are shown in Fig. 5C. The specific AK activities, L-arginine production, biomass, L-arginine yield, lysine and isoleucine formation of the Cc5-800 and Cc5*lysC* strains are listed in Table S12. It can be seen that the extremely low activity of AK had a strong negative effect on cell growth. The strain Cc5*lysC*-30 showed the highest L-arginine production and yield, and the levels of lysine and isoleucine were very low.

In *C. glutamicum*, the proline is synthesized in four reactions from glutamate, and the synthesis of 1 mol of proline requires 2 mol of NADPH (Fig. 1). Glutamate kinase (GK) encoded by *proB*, catalyzes the conversion of glutamate to γ**-glutamyl phosphate, the first step in proline biosynthesis^22^. To eliminate the proline synthesis in the Cc5*lysC*-30 strain, the GK was inactivated by the deletion of *proB* gene in the Cc5*lysC*-30 strain, resulting strain Cc6. This strain showed higher L-arginine production and yield and the proline was undetectable in fermentation broth (Table S13). Fed-batch fermentation of the Cc6 strain allowed production of 87.3 g L^−1^ L-arginine with a productivity of 1.21 g L^−1^ h^−1^ and yield of 0.431 g g^−1^ (Fig. 3 and Table 1). The levels of lysine and isoleucine were very low and the proline was undetectable (Table 2). Therefore, elimination of the competition of byproducts synthesis for carbon flux and cofactor is effective to increase the L-arginine production and yield on glucose.

### Pilot-scale fermentation of the Cc0 and Cc6 strains

**Figure 6:**
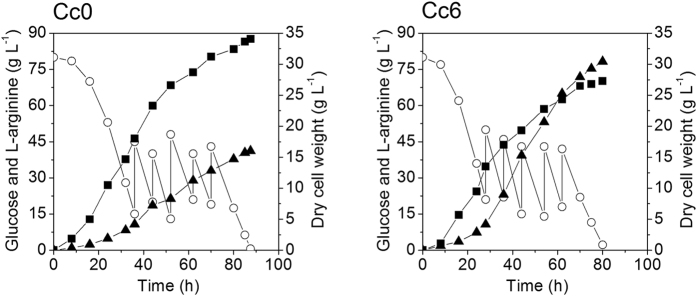
Time course of pilot-scale L-arginine fed-batch fermentations of Cc0 and Cc6 strains. Signal denotes: L-arginine (filled triangles), dry cell weight (filled squares), glucose (open circles).

The production performance of Cc0 and Cc6 strains were investigated in pilot-scale fermentations. For this purpose, a medium based on corn steep liquor was used, because corn steep liquor is typically applied for industrial amino acid production, and the fermentations were performed in a 1000 L bioreactor (Fig. S1). Figure 6 shows the time profiles of pilot-scale fermentations of Cc0 and Cc6 strains. Pilot-scale fermentation of the Cc6 strain resulted in 78.4 g L^−1^ of L-arginine with a productivity of 0.98 g L^−1^ h^−1^ and yield of 0.387 g g^−1^ glucose. In comparison, pilot-scale fermentation of the Cc0 strain resulted in 40.3 g L^−1^ of L-arginine with a productivity of 0.46 g L^−1^ h^−1^ and yield of 0.193 g g^−1^ glucose. The results showed that the final strain developed by systems pathway engineering also allowed efficient L-arginine production under pilot-scale fermentation conditions. According to the production data from enterprise, if the fermentation performance of the Cc6 strain can be realized under commercial scale production conditions, the cost of L-arginine production by fermentation will be lower than the price of L-arginine produced by keratin hydrolysis method (Table S14). Thus, L-arginine production by fermentation of the final strain constructed in this paper has great potential to substitute the keratin hydrolysis method in China.

## Conclusions

In this work, we performed systems pathway engineering of *C. crenatum* to regulate the carbon flux of L-arginine synthesis pathway, central carbon core metabolism and byproducts formation, and optimize the glucose uptake globally based on publications and the analysis of the unique fermentation characteristics of our strains such as glucose consumption, byproducts formation and intracellular NADPH level. All genetic modifications were introduced into the genome (Fig. 1) such that the resulting strains are stable and the L-arginine production by these strains does not require the use of selection markers. The concentration, yield and productivity of L-arginine fermentation were enhanced step by step through the amplification of L-arginine biosynthetic flux, regulation of central carbon core metabolism, improvement of glucose uptake and minimization of carbon and cofactor loss. Pilot-scale fermentation of the final strain resulted in 78.4 g L^−1^ of L-arginine with a yield of 0.387 g g^−1^ glucose and productivity of 0.98 g L^−1^ h^−1^. The results indicated that L-arginine production by the final strain constructed in this paper has great potential to substitute the keratin hydrolysis method. And the engineering strategy used in this work may be used to construct efficient cell factories for the green production of the other industrially important chemicals.

## Methods

### Strains, plasmids, and culture conditions

*C. crenatum* SYPA5-5 (subspecies of *C. glutamicum*), deposited as CGMCC 0890, the L-arginine producer obtained by multiple random mutagenesis was used as the parent strain for strain engineering^5^,^15^,^33^. For genetic engineering work, *Escherichia coli* strain JM109 and plasmid pK18*mobsacB*^34^ were applied. All bacterial strains and plasmids used in this study as well as their relevant characteristics are listed in Table S15 and Table S16. The *C. crenatum* SYPA5-5 and its recombinant derivatives were routinely cultivated aerobically at 30 °C in LBG medium (LB medium supplemented with 5 g L^−1^ glucose). For recombinant DNA work, *E. coli* JM109 was cultivated at 37 °C in LB medium. Where appropriate, kanamycin (25 mg L^−1^ for *C. crenatum* strains, 50 mg L^−1^ for *E. coli* JM109) were added to the medium.

### Recombinant DNA Work for Plasmid and Strain Construction

All modifications were introduced into the genome using the homologous *sacB* recombination system^34^. All DNA manipulations were performed by standard procedures. For strain construction, transformation of *C. glutamicum* strains via electroporation, Modified genotypes in all strains were confirmed by PCR and DNA sequencing. The detailed procedures for genetic engineering and primer sequences (Table S17) used in this study are supplied in the supplementary section.

### Microbial production of L-arginine

Batch fermentations were performed in shake flasks. Fed-batch fermentations were carried out in 5 L stirred fermenters (BIOTECH-5BG, Baoxing Co., China). Pilot-scale fermentations were performed in 1000 L bioreactor. The detailed fermentation conditions and procedures are supplied in the supplementary section.

### Quantification of substrates and products

Dry cell weight (DCW) was determined from a calibration curve of known DCW and the corresponding optical density at 562 nm (1 OD_562_ = 0.375 g L^−1^ DCW) using a spectrophotometer (UNICO^TM^-UV2000, Shanghai, China). For quantification of substrate consumption and product formation, 2 mL samples of the culture were harvested and spinned down (10,000 × *g*, 10 min, and 4 °C). The resulting supernatants were used for determination of glucose, amino acids, and organic acids concentrations in the culture fluid. The glucose was measured enzymatically using using a bioanalyzer (SBA-40C, Shandong, China). Amino acids (L-arginine, L-glutamate, L-lysine, L-isoleucine, L-threonine, L-leucine and L-valine, etc.) were measured by high-pressure liquid chromatography on Agilent 1100 LC system (Agilent Technologies, Waldbronn, Germany), following the procedures reported by Xu^15^. Organic acids (acetate, lactate, 2-oxoglutarate, succinate, fumarate, oxaloacetate, etc.) were also measured by high-pressure liquid chromatography on Agilent 1100 LC system, following the procedures reported by Wieschalka^35^. Each assay was replicated three times.

### Preparation of crude extracts and enzyme assays

For enzyme activity measurements, *C. crenatum* cells were harvested at the exponential phase during the batch fermentations in shake flasks by centrifugation (5,000 *g* for 10 min at 4 °C) and washed twice with 40 mL 100 mM Tris-HCl buffer (pH 8.0). The cells were resuspended in 5 mL of the same buffer, and the cell disruption was achieved by sonication. Cell debris was removed by centrifugation at 4 °C (10,000 *g* for 10 min), and the supernatant was stored on ice until further use. The protein concentration was determined by Bradford method^36^. Activities of phosphofructokinase^19^, phosphoglucoisomerase^19^, pyruvate carboxylase^37^, citrate synthase^38^, isocitrate dehydrogenase^30^, glutamate dehydrogenase^39^, α-ketoglutarate dehydrogenase^40^, aspartokinase^41^, glutamate kinase^42^ were determined as described previously. Each assay was replicated three times.

### NADPH measurement and RNA preparation and transcriptional analysis

For NADPH measurements, *C. crenatum* cells were harvested at the exponential phase during the batch fermentations in shake flasks by centrifugation (5,000 *g* for 10 min at 4 °C). The intracellular concentrations of NADPH were measured using a NADP/NADPH quantitation kit (BioVision, Inc., Milpitas, CA) according to the manufacturer’s instructions.

Total RNA was extracted from *C. crenatum* cells at the exponential phase during the batch fermentations in shake flasks using the RNAiso Plus reagent (Takara, Dalian, China). The cDNA was synthesized with a PrimeScript RT reagent kit (Takara, Dalian, China). Real-time PCR (RT-PCR) was performed on a Bio-Rad CFX96 Touch Real-Time PCR Detection System (Bio-Rad Hercules, CA) using the SYBR *Premix Ex Taq*^TM^ II (Takara, Dalian, China). The 16S rRNA gene was used as the internal standard^16^. The primer sequences used in RT-PCR are supplied in the supplementary section (Table S18). Each assay was replicated three times.

## Additional Information

**How to cite this article**: Man, Z. *et al*. Systems pathway engineering of *Corynebacterium crenatum* for improved L-arginine production. *Sci. Rep*. **6**, 28629; doi: 10.1038/srep28629 (2016).

## Supplementary Material

Supplementary Information

## Figures and Tables

**Table 1:** L-arginine fed-batch fermentation parameters of different strains.

Strain	Arginine production (g L^−1^)	Arginine yield on glucose (g g^−1^)	Dry cell weight (DCW) (g L^−1^)	Specific arginine yield (g g^−1^ DCW)	Productivity (g L^−1^ h^−1^)
Cc0	41.2 ± 2.3	0.202 ± 0.013	27.9 ± 1.0	1.48 ± 0.11	0.49 ± 0.03
Cc1	53.2 ± 1.9	0.256 ± 0.016	25.9 ± 0.9	2.05 ± 0.15	0.71 ± 0.05
Cc2-6500	66.4 ± 1.8	0.325 ± 0.015	25.0 ± 1.3	2.66 ± 0.13	0.69 ± 0.04
Cc3	61.0 ± 1.5	0.294 ± 0.018	28.7 ± 1.3	2.13 ± 0.10	0.93 ± 0.06
Cc4	68.6 ± 2.1	0.336 ± 0.016	31.2 ± 1.2	2.20 ± 0.14	1.08 ± 0.05
Cc5-800	76.8 ± 2.5	0.372 ± 0.019	26.3 ± 1.2	2.92 ± 0.17	1.12 ± 0.07
Cc6	87.3 ± 2.6	0.431 ± 0.021	23.4 ± 1.1	3.73 ± 0.22	1.21 ± 0.06

Cc0: parent strain *C. crenatum* SYPA5-5; Cc1: Cc0+replacement of the natural *argB* gene by mutated *argB*_M3_ gene, and replacement of the natural promoters of the *argCJBDFR* and *argGH* operons by the *eftu* promoter; Cc2-6500: Cc1+replacement of the natural RBS (11290 au of activity) of *pgi* gene by weaker RBS (6500 au of activity); Cc3: Cc2-6500+replacement of the natural promoters of the *ptsG* gene, *iolT1* gene and *ppgk* gene by the *sod* promoter; Cc4: Cc3+replacement of the start codon GTG by ATG in the *pyc* gene and implementation of an additional copy of *gltA* gene on chromosome; Cc5-800: Cc4+implementation of an additional copy of *icd* gene and *gdh* gene on chromosome, respectively, and replacement of the natural RBS (1613 au of activity) of *odhA* gene by weaker RBS (800 au of activity); Cc6: Cc5-800+replacement of the natural RBS (131 au of activity) of *lysC* gene by weaker RBS (30 au of activity) and deletion of *proB* gene. SDs based on three biologically independent experiments.

**Table 2:** The main byproducts formation in L-arginine fed-batch fermentations of different strains.

Strain	Lysine (g L^−1^)	Isoleucine (g L^−1^)	Proline (g L^−1^)	Acetate (g L^−1^)	Lactate (g L^−1^)
Cc0	2.53 ± 0.38	2.24 ± 0.25	1.16 ± 0.21	0.87 ± 0.21	1.83 ± 0.13
Cc1	1.94 ± 0.29	1.82 ± 0.18	0.57 ± 0.16	0.69 ± 0.14	1.42 ± 0.17
Cc2-6500	4.12 ± 0.64	3.06 ± 0.47	1.38 ± 0.25	0.36 ± 0.08	0.54 ± 0.08
Cc3	4.36 ± 0.55	2.85 ± 0.36	1.56 ± 0.27	1.84 ± 0.39	4.32 ± 0.87
Cc4	4.63 ± 0.59	3.18 ± 0.43	1.71 ± 0.23	0.49 ± 0.08	0.67 ± 0.06
Cc5-800	3.74 ± 0.46	2.83 ± 0.38	2.12 ± 0.30	0.31 ± 0.07	0.42 ± 0.05
Cc6	0.61 ± 0.17	0.39 ± 0.12	Not detected	0.23 ± 0.05	0.37 ± 0.05

Cc0: parent strain *C. crenatum* SYPA5-5; Cc1: Cc0+replacement of the natural *argB* gene by mutated *argB*_M3_ gene, and replacement of the natural promoters of the *argCJBDFR* and *argGH* operons by the *eftu* promoter; Cc2-6500: Cc1+replacement of the natural RBS (11290 au of activity) of *pgi* gene by weaker RBS (6500 au of activity); Cc3: Cc2-6500+replacement of the natural promoters of the *ptsG* gene, *iolT1* gene and *ppgk* gene by the *sod* promoter; Cc4: Cc3+replacement of the start codon GTG by ATG in the *pyc* gene and implementation of an additional copy of *gltA* gene on chromosome; Cc5-800: Cc4+implementation of an additional copy of *icd* gene and *gdh* gene on chromosome, respectively, and replacement of the natural RBS (1613 au of activity) of *odhA* gene by weaker RBS (800 au of activity); Cc6: Cc5-800+replacement of the natural RBS (131 au of activity) of *lysC* gene by weaker RBS (30 au of activity) and deletion of *proB* gene. The contents of the other byproducts were very low (≤0.50 g L^−1^). SDs based on three biologically independent experiments.
