# Supplementary material for: Systems pathway engineering of Corynebacterium crenatum for improved L-arginine production
Source: Sci Rep. 2016 Jun 24;6:28629. doi: 10.1038/srep28629 (PMC4919616; doi:10.1038/srep28629)
Supplement: Supplementary Information [file srep28629-s1.pdf]

Systems pathway engineering of *Corynebacterium crenatum* for improved L-arginine production

SUPPLEMENTARY INFORMATION

Zaiwei Man[1], Meijuan Xu[1], Zhiming Rao[1,2]*, Jing Guo[1], Taowei Yang[1], Xian Zhang[1] & Zhenghong Xu[3]

[1]The Key Laboratory of Industrial Biotechnology, Ministry of Education, School of Biotechnology, Jiangnan University, Wuxi 214122, China

[2]State Key Laboratory of Food Science and Technology, Jiangnan University, Wuxi 214122, China

[3]School of Pharmaceutical Sciences, Jiangnan University, Wuxi 214122, China

*Corresponding author: Zhiming Rao, Email: raozhm@jiangnan.edu.cn, Phone: +86-510-85916881

Plasmid and strain constructions[1,2]

For construction of pK18-*argB*$_{M3}$, the *argB*$_{M3}$ gene was amplified by PCR with primers argB-1 and argB-2, from plasmid pJCB$_{M3}$[3]. In the next step, the amplified DNA fragment was digested with the restriction enzymes *EcoR*I and *Sal*I and ligated into the equally digested vector pK18, resulting plasmid pK18-*argB*$_{M3}$. This plasmid was used to transform Cc0 using electroporation, which results in the first recombination. The selected colonies were subjected to the second recombination on the plate with LBG medium containing 10% (w/v) sucrose. The modified genotype was investigated by PCR analysis and determination of enzyme activity. A positively identified mutant, in the following referred to as CcMB, was used for subsequent genetic modifications.

For construction of pK18-P$_{eftu}$*argCJBDFR*, the DNA fragment upstream of *argC* gene, the sequence of *eftu*-promoter and the upstream region of *argC* gene were amplified using the primer combinations argC-1/argC-2, Peftu-1/ Peftu-2, and argC-3/argC-4 from genomic DNA of *C. crenatum* SYPA5-5 in PCR1, PCR2 and PCR3, respectively. In PCR4, the amplified DNA-fragment upstream of *argC* gene from PCR1 and the amplified DNA-fragment of the *eftu*-promoter from PCR2 were fused in an overlap-extension PCR using primers argC-1 and Peftu-2. In PCR5, the amplified DNA fragment of the upstream region of *argC* gene from PCR3 and the amplified DNA fragment from PCR4 were fused in an overlap-extension PCR using primers argC-1 and argC-4. The resulting DNA fragment contained the DNA fragment upstream of *argC* gene, the sequence of *eftu*-promoter and the upstream region of *argC* gene from upstream to downstream, as well as recognition sites *EcoR*I and *Hind*III which were added by argC-1 and argC-4, respectively. This fragment was digested with the restriction enzymes *EcoR*I and *Hind*III and ligated into the equally digested vector pK18, resulting plasmid pK18-P$_{eftu}$*argCJBDFR*. This plasmid was used to transform CcMB using electroporation, which results in the first recombination. The selected colonies were subjected to the second recombination on the plate with LBG medium containing 10% (w/v) sucrose. The modified genotype was investigated by PCR analysis. A positively identified mutant, in the following referred to as CcMB-P$_{eftu}$*argCJBDFR*, was used for subsequent genetic modifications.

For construction of pK18-P$_{eftu}$*argGH*, the DNA fragment upstream of *argG* gene, the sequence of *eftu*-promoter and the upstream region of *argG* gene were amplified using the primer combinations argG-1/argG-2, Peftu-1/ Peftu-2, and argG-3/argG-4

from genomic DNA of *C. crenatum* SYPA5-5 in PCR1, PCR2 and PCR3, respectively. In PCR4, the amplified DNA-fragment upstream of *argG* gene from PCR1 and the amplified DNA-fragment of the *eftu*-promoter from PCR2 were fused in an overlap-extension PCR using primers argG-1 and Peftu-2. In PCR5, the amplified DNA fragment of the upstream region of *argG* gene from PCR3 and the amplified DNA fragment from PCR4 were fused in an overlap-extension PCR using primers argG-1 and argG-4. The resulting DNA fragment contained the DNA fragment upstream of *argG* gene, the sequence of *eftu*-promoter and the upstream region of *argG* gene from upstream to downstream, as well as recognition sites *EcoR*I and *Hind*III which were added by argG-1 and argG-4, respectively. This fragment was digested with the restriction enzymes *EcoR*I and *Hind*III and ligated into the equally digested vector pK18, resulting plasmid pK18-P_{eftu}*argGH*. This plasmid was used to transform CcMB-P_{eftu}*argCJBDFR* using electroporation, which results in the first recombination. The selected colonies were subjected to the second recombination on the plate with LBG medium containing 10% (w/v) sucrose. The modified genotype was investigated by PCR analysis. A positively identified mutant, in the following referred to as Cc1, was used for subsequent genetic modifications.

For construction of pK18-2*pfk*, the *pfk* gene was amplified by PCR with primers pfk-1, pfk-2, pfk-3 and pfk-4. In PCR1 and PCR2 the complete *pfk* sequence was amplified together with flanking regions upstream and downstream of the *pfk* gene using the primer combinations pfk-1/pfk-2 and pfk-3/pfk-4, respectively, from genomic DNA of *C. crenatum* SYPA5-5. In the next step, the amplified DNA fragments were fused in an overlap-extension PCR using primers pfk-1 and pfk-4. The resulting DNA fragment contained two complete *pfk* genes each flanked with upstream and downstream sequences, as well as recognition sites *EcoR*I and *BamH*I which were added by pfk-1 and pfk-4, respectively. This fragment was digested with the restriction enzymes *EcoR*I and *BamH*I and ligated into the equally digested vector pK18, resulting plasmid pK18-2*pfk*. This plasmid was used to transform Cc1 using electroporation, which results in the first recombination. The selected colonies were subjected to the second recombination on the plate with LBG medium containing 10% (w/v) sucrose. The modified genotype was investigated by PCR analysis and determination of enzyme activity. A positively identified mutant, in the following referred to as Cc1-2*pfk*, was used for subsequent genetic modifications.

For construction of pK18-rbs*pgi*, the DNA fragment upstream of *pgi* gene and

the upstream region of *pgi* gene were amplified using the primer combinations pgi-1/pgi-2 and pgi-3/pgi-4, respectively, from genomic DNA of *C. crenatum* SYPA5-5. In the next step, the amplified DNA fragments were fused in an overlap-extension PCR using primers pgi-1 and pgi-4. In the resulting DNA fragments the sequence of natural RBS of *pgi* gene was replaced by the sequences of synthetic RBSs with strengths of 4000 au, 5000 au, 6500 au and 8500 au which were added by the primer pgi-3, respectively, as well as two recognition sites *EcoR*I and *Hind*III which were added by pgi-1 and pgi-4, respectively. These fragments were digested with the restriction enzymes *EcoR*I and *Hind*III and ligated into the equally digested vector pK18, resulting plasmids pK18-4000rbs*pgi*, pK18-5000rbs*pgi*, pK18-6500rbs*pgi*, pK18-8500rbs*pgi*, respectively. These plasmids were used to transform Cc1 using electroporation, which result in the first recombination. The selected colonies were subjected to the second recombination on the plates with LBG medium containing 10% (w/v) sucrose. The modified genotypes were investigated by PCR and DNA sequencing analysis and determination of enzyme activity. A positively identified mutant, in the following referred to as Cc2, was used for subsequent genetic modifications.

For construction of pK18-P_{sod}*ptsG*, the DNA fragment upstream of *ptsG* gene, the sequence of *sod*-promoter and the upstream region of *ptsG* gene were amplified using the primer combinations ptsG-1/ptsG-2, Psod-1/ Psod-2, and ptsG-3/ptsG-4 from genomic DNA of *C. crenatum* SYPA5-5 in PCR1, PCR2 and PCR3, respectively. In PCR4, the amplified DNA-fragment upstream of *ptsG* gene from PCR1 and the amplified DNA-fragment of the *sod*-promoter from PCR2 were fused in an overlap-extension PCR using primers ptsG-1 and Psod-2. In PCR5, the amplified DNA fragment of the upstream region of *ptsG* gene from PCR3 and the amplified DNA fragment from PCR4 were fused in an overlap-extension PCR using primers ptsG-1 and ptsG-4. The resulting DNA fragment contained the DNA fragment upstream of *ptsG* gene, the sequence of *sod*-promoter and the upstream region of *ptsG* gene from upstream to downstream, as well as recognition sites *EcoR*I and *Hind*III which were added by ptsG-1 and ptsG-4, respectively. This fragment was digested with the restriction enzymes *EcoR*I and *Hind*III and ligated into the equally digested vector pK18, resulting plasmid pK18-P_{sod}*ptsG*. This plasmid was used to transform Cc2-6500 using electroporation, which results in the first recombination. The selected colonies were subjected to the second recombination on the plate with LBG medium

containing 10% (w/v) sucrose. The modified genotype was investigated by PCR analysis. A positively identified mutant, in the following referred to as Cc2-G_{sod}, was used for subsequent genetic modifications.

For construction of pK18-P_{sod}*iolT1*, the DNA fragment upstream of *iolT1* gene, the sequence of *sod*-promoter and the upstream region of *iolT1* gene were amplified using the primer combinations iolT1-1/ iolT1-2, Psod-1/ Psod-2, and iolT1-3/ iolT1-4 from genomic DNA of *C. crenatum* SYPA5-5 in PCR1, PCR2 and PCR3, respectively. In PCR4, the amplified DNA-fragment upstream of *iolT1* gene from PCR1 and the amplified DNA-fragment of the *sod*-promoter from PCR2 were fused in an overlap-extension PCR using primers iolT1-1 and Psod-2. In PCR5, the amplified DNA fragment of the upstream region of *iolT1* gene from PCR3 and the amplified DNA fragment from PCR4 were fused in an overlap-extension PCR using primers iolT1-1 and iolT1-4. The resulting DNA fragment contained the DNA fragment upstream of *iolT1* gene, the sequence of *sod*-promoter and the upstream region of *iolT1* gene from upstream to downstream, as well as recognition sites *EcoR*I and *Hind*III which were added by iolT1-1 and iolT1-4, respectively. This fragment was digested with the restriction enzymes *EcoR*I and *Hind*III and ligated into the equally digested vector pK18, resulting plasmid pK18-P_{sod}*iolT1*. This plasmid was used to transform Cc2-G_{sod} using electroporation, which results in the first recombination. The selected colonies were subjected to the second recombination on the plate with LBG medium containing 10% (w/v) sucrose. The modified genotype was investigated by PCR analysis. A positively identified mutant, in the following referred to as Cc2-G_{sod}-P_{sod}*iolT1*, was used for subsequent genetic modifications.

For construction of pK18-P_{sod}*ppgk*, the DNA fragment upstream of *ppgk* gene, the sequence of *sod*-promoter and the upstream region of *ppgk* gene were amplified using the primer combinations ppgk-1/ppgk-2, Psod-1/ Psod-2, and ppgk-3/ppgk-4 from genomic DNA of *C. crenatum* SYPA5-5 in PCR1, PCR2 and PCR3, respectively. In PCR4, the amplified DNA-fragment upstream of *ppgk* gene from PCR1 and the amplified DNA-fragment of the *sod*-promoter from PCR2 were fused in an overlap-extension PCR using primers ppgk-1 and Psod-2. In PCR5, the amplified DNA fragment of the upstream region of *ppgk* gene from PCR3 and the amplified DNA fragment from PCR4 were fused in an overlap-extension PCR using primers ppgk-1 and ppgk-4. The resulting DNA fragment contained the DNA fragment upstream of *ppgk* gene, the sequence of *sod*-promoter and the upstream region of *ppgk*

gene from upstream to downstream, as well as recognition sites *EcoR*I and *Hind*III which were added by ppgk-1 and ppgk-4, respectively. This fragment was digested with the restriction enzymes *EcoR*I and *Hind*III and ligated into the equally digested vector pK18, resulting plasmid pK18-P_{sod}*ppgk*. This plasmid was used to transform Cc2-G_{sod}-P_{sod}*iolT1* using electroporation, which results in the first recombination. The selected colonies were subjected to the second recombination on the plate with LBG medium containing 10% (w/v) sucrose. The modified genotype was investigated by PCR analysis. A positively identified mutant, in the following referred to as Cc3, was used for subsequent genetic modifications.

For construction of pK18-ATG*pyc*, the DNA fragment upstream of *pyc* gene and the upstream region of *pyc* gene were amplified using the primer combinations pyc-1/pyc-2 and pyc-3/pyc-4, respectively, from genomic DNA of *C. crenatum* SYPA5-5. In the next step, the amplified DNA fragments were fused in an overlap-extension PCR using primers pyc-1 and pyc-4. In the resulting DNA fragments the natural start codon GTG of *pyc* gene was replaced by ATG through the primer pyc-3, as well as two recognition sites *EcoR*I and *Hind*III which were added by pyc-1 and pyc-4, respectively. This fragment was digested with the restriction enzymes *EcoR*I and *Hind*III and ligated into the equally digested vector pK18, resulting plasmid pK18-ATG*pyc*. This plasmid was used to transform Cc3 using electroporation, which result in the first recombination. The selected colonies were subjected to the second recombination on the plates with LBG medium containing 10% (w/v) sucrose. The modified genotypes were investigated by PCR and DNA sequencing analysis and determination of enzyme activity. A positively identified mutant, in the following referred to as Cc3-pyc_{G1A}, was used for subsequent genetic modifications.

For construction of pK18-2*gltA*, the *gltA* gene was amplified by PCR with primers gltA-1, gltA-2, gltA-3 and gltA-4. In PCR1 and PCR2 the complete *gltA* sequence was amplified together with flanking regions upstream and downstream of the *gltA* gene using the primer combinations gltA-1/gltA-2 and gltA-3/gltA-4, respectively, from genomic DNA of *C. crenatum* SYPA5-5. In the next step, the amplified DNA fragments were fused in an overlap-extension PCR using primers gltA-1 and gltA-4. The resulting DNA fragment contained two complete *gltA* genes each flanked with upstream and downstream sequences, as well as recognition sites *EcoR*I and *Hind*III which were added by gltA-1 and gltA-4, respectively. This

fragment was digested with the restriction enzymes *Eco*RI and *Hind*III and ligated into the equally digested vector pK18, resulting plasmid pK18-2*gltA*. This plasmid was used to transform Cc3-*pyc*$_{G1A}$ using electroporation, which results in the first recombination. The selected colonies were subjected to the second recombination on the plate with LBG medium containing 10% (w/v) sucrose. The modified genotype was investigated by PCR analysis and determination of enzyme activity. A positively identified mutant, in the following referred to as Cc4, was used for subsequent genetic modifications.

For construction of pK18-2*icd*, the *icd* gene was amplified by PCR with primers icd-1, icd-2, icd-3 and icd-4. In PCR1 and PCR2 the complete *icd* sequence was amplified together with flanking regions upstream and downstream of the *icd* gene using the primer combinations icd-1/icd-2 and icd-3/icd-4, respectively, from genomic DNA of *C. crenatum* SYPA5-5. In the next step, the amplified DNA fragments were fused in an overlap-extension PCR using primers icd-1 and icd-4. The resulting DNA fragment contained two complete *icd* genes each flanked with upstream and downstream sequences as well as recognition sites *Eco*RI and *Xba*I which were added by icd-1 and icd-4, respectively. This fragment was digested with the restriction enzymes *Eco*RI and *Xba*I and ligated into the equally digested vector pK18, resulting plasmid pK18-2*icd*. This plasmid was used to transform Cc4 using electroporation, which results in the first recombination. The selected colonies were subjected to the second recombination on the plate with LBG medium containing 10% (w/v) sucrose. The modified genotype was investigated by PCR analysis and determination of enzyme activity. A positively identified mutant, in the following referred to as Cc4-2*icd*, was used for subsequent genetic modifications.

For construction of pK18-2*gdh*, the *gdh* gene was amplified by PCR with primers gdh-1, gdh-2, gdh-3 and gdh-4. In PCR1 and PCR2 the complete *gdh* sequence was amplified together with flanking regions upstream and downstream of the *gdh* gene using the primer combinations gdh-1/gdh-2 and gdh-3/gdh-4, respectively, from genomic DNA of *C. crenatum* SYPA5-5. In the next step, the amplified DNA fragments were fused in an overlap-extension PCR using primers gdh-1 and gdh-4. The resulting DNA fragment contained two complete *gdh* genes each flanked with upstream and downstream sequences as well as recognition sites *Eco*RI and *Hind*III which were added by gdh-1 and gdh-4, respectively. This fragment was digested with the restriction enzymes *Eco*RI and *Hind*III and ligated into the

equally digested vector pK18, resulting plasmid pK18-2*gdh*. This plasmid was used to transform Cc4-2*icd* using electroporation, which results in the first recombination. The selected colonies were subjected to the second recombination on the plate with LBG medium containing 10% (w/v) sucrose. The modified genotype was investigated by PCR analysis and determination of enzyme activity. A positively identified mutant, in the following referred to as Cc4-2*icd*-2*gdh*, was used for subsequent genetic modifications.

For construction of pK18-rbs*odhA*, the DNA fragment upstream of *odhA* gene and the upstream region of *odhA* gene were amplified using the primer combinations odhA-1/odhA-2 and odhA-3/odhA-4, respectively, from genomic DNA of *C. crenatum* SYPA5-5. In the next step, the amplified DNA fragments were fused in an overlap-extension PCR using primers odhA-1 and odhA-4. In the resulting DNA fragments the sequence of natural RBS of *odhA* gene was replaced by the sequences of synthetic RBSs with strengths of 200 au, 500 au, 800 au and 1200 au which were added by the primer odhA-3, respectively, as well as two recognition sites *EcoR*I and *BamH*I which were added by odhA-1 and odhA-4, respectively. These fragments were digested with the restriction enzymes *EcoR*I and *BamH*I and ligated into the equally digested vector pK18, resulting plasmids pK18-200rbs*odhA*, pK18-500rbs*odhA*, pK18-800rbs*odhA*, pK18-1200rbs*odhA*, respectively. These plasmids were used to transform Cc4-2*icd*-2*gdh* using electroporation, which result in the first recombination. The selected colonies were subjected to the second recombination on the plates with LBG medium containing 10% (w/v) sucrose. The modified genotypes were investigated by PCR and DNA sequencing analysis and determination of enzyme activity. A positively identified mutant, in the following referred to as Cc5, was used for subsequent genetic modifications.

For construction of pK18-rbs*lysC*, the DNA fragment upstream of *lysC* gene and the upstream region of *lysC* gene were amplified using the primer combinations lysC-1/lysC-2 and lysC-3/lysC-4, respectively, from genomic DNA of *C. crenatum* SYPA5-5. In the next step, the amplified DNA fragments were fused in an overlap-extension PCR using primers lysC-1 and lysC-4. In the resulting DNA fragments the sequence of natural RBS of *lysC* gene was replaced by the sequences of synthetic RBSs with strengths of 15 au, 30 au, 60 au and 100 au which were added by the primer lysC-3, respectively, as well as two recognition sites *EcoR*I and *Hind*III which were added by lysC-1 and lysC-4, respectively. These fragments were digested

with the restriction enzymes *EcoR*I and *Hind*III and ligated into the equally digested vector pK18, resulting plasmids pK18-15rbs*lysC*, pK18-30rbs*lysC*, pK18-60rbs*lysC*, pK18-100rbs*lysC*, respectively. These plasmids were used to transform Cc5-800 using electroporation, which result in the first recombination. The selected colonies were subjected to the second recombination on the plates with LBG medium containing 10% (w/v) sucrose. The modified genotypes were investigated by PCR and DNA sequencing analysis and determination of enzyme activity. A positively identified mutant, in the following referred to as Cc5*lysC*, was used for subsequent genetic modifications.

For construction of pK18-Δ*proB*, the upstream and downstream regions of *proB* gene were amplified using the primer combinations proB-1/proB-2 and proB-3/proB-4, respectively, from genomic DNA of *C. crenatum* SYPA5-5. In the next step, the amplified DNA fragments were fused in an overlap-extension PCR using primers proB-1 and proB-4. The resulting DNA fragment contained an incomplete *proB* gene, lacking about 500 nucleotides, as well as two recognition sites *EcoR*I and *Hind*III which were added by proB-1 and proB-4, respectively. This fragment was digested with the restriction enzymes *EcoR*I and *Hind*III and ligated into the equally digested vector pK18, resulting plasmid pK18-Δ*proB*. This plasmid was used to transform Cc5*lysC*-30 using electroporation, which results in the first recombination. The selected colonies were subjected to the second recombination on the plate with LBG medium containing 10% (w/v) sucrose. The modified genotype was investigated by PCR analysis and determination of enzyme activity. A positively identified mutant, in the following referred to as Cc6.

Microbial production of L-arginine

Batch fermentations in shake flasks were performed as follows. A stock culture was maintained and revived on agar slants containing 10 g L^{-1} peptone, 10 g L^{-1} beef extract, 5 g L^{-1} yeast extract, 5 g L^{-1} NaCl, 20 g L^{-1} agar. One loop of colonies from agar slants was inoculated into 20 mL seed medium containing 30 g L^{-1} glucose, 20 g L^{-1} yeast extract, 20 g L^{-1} $(NH_4)_2SO_4$, 1 g L^{-1} KH_2PO_4, 0.5 g L^{-1} $MgSO_4 \cdot 7H_2O$, and cultured at 30 ^oC and 200 rpm for 15 h in 250 mL shake flasks. Then, 1 mL seed culture was transferred into 250 mL shake flasks containing 25 mL fermentation medium, and cultured at 30 ^oC and 200 rpm. The fermentation medium contained 100 g L^{-1} glucose, 10 g L^{-1} yeast extract, 20 g L^{-1} $(NH_4)_2SO_4$, 1.5 g L^{-1} KH_2PO_4, 0.5 g L^{-1}

$MgSO_4 \cdot 7H_2O$, 0.02 g L^{-1} $FeSO_4 \cdot 7H_2O$, 0.02 g L^{-1} $MnSO_4$, 30 g L^{-1} $CaCO_3$. The batch cultures were performed in triplicate.

Fed-batch fermentations were carried out in 5 L stirred fermenters (BIOTECH-5BG, Baoxing Co., China). A stock culture was maintained and revived on agar slants containing 10 g L^{-1} peptone, 10 g L^{-1} beef extract, 5 g L^{-1} yeast extract, 5 g L^{-1} NaCl, 20 g L^{-1} agar. One loop of colonies from agar slants was inoculated into 50 mL seed medium containing 30 g L^{-1} glucose, 20 g L^{-1} yeast extract, 20 g L^{-1} $(NH_4)_2SO_4$, 1 g L^{-1} KH_2PO_4, 0.5 g L^{-1} $MgSO_4 \cdot 7H_2O$, and cultured at 30 ^oC and 200 rpm for 20 h in 500 mL shake flasks. The seed culture (100 mL) was transferred into the 5 L stirred fermenter. Fermentation was started with a volume of 1.5 L. The fermentation medium contained 80 g L^{-1} glucose, 10 g L^{-1} yeast extract, 20 g L^{-1} $(NH_4)_2SO_4$, 1.5 g L^{-1} KH_2PO_4, 0.5 g L^{-1} $MgSO_4 \cdot 7H_2O$, 0.02 g L^{-1} $FeSO_4 \cdot 7H_2O$, 0.02 g L^{-1} $MnSO_4$. The feed medium contained 500 g L^{-1} glucose, 10 g L^{-1} yeast extract, 20 g L^{-1} $(NH_4)_2SO_4$. The feed medium was fed into the fermenter when the residual concentration of glucose became below 20 g L^{-1}. The fed-batch fermentation was performed at 30 ^oC; pH was maintained at 7.0 by automatically adding 30% NH_4OH solution; the agitation speed was controlled at 600 rpm and the air flow rate was maintained at 3.0 L min^{-1}.

Pilot-scale fermentations were performed as follows. Corn starch hydrolysate and corn steep liquor were used as carbon source and nitrogen source, respectively. The first seed culture was cultivated in first seed medium containing 40 g L^{-1} glucose (supplied by corn starch hydrolysate), 40 g L^{-1} corn steep liquor, 20 g L^{-1} $(NH_4)_2SO_4$, 1 g L^{-1} KH_2PO_4, 0.5 g L^{-1} $MgSO_4 \cdot 7H_2O$, at 30 ^oC for 20 h in 2 L shake flasks. Then, the first seed culture (3.0 L) was transferred into the 100 L stirred fermenter containing 50 L second seed medium, and cultured at 30 ^oC and 350 rpm for 20 h. The second seed medium contained 40 g L^{-1} glucose (supplied by corn starch hydrolysate), 30 g L^{-1} corn steep liquor, 20 g L^{-1} $(NH_4)_2SO_4$, 1 g L^{-1} KH_2PO_4, 0.5 g L^{-1} $MgSO_4 \cdot 7H_2O$. Finally, this second seed culture was inoculated into a 1000 L bioreactor containing 500 L fermentation medium. The fermentation medium contained 80 g L^{-1} glucose (supplied by corn starch hydrolysate), 25 g L^{-1} corn steep liquor, 20 g L^{-1} $(NH_4)_2SO_4$, 1.5 g L^{-1} KH_2PO_4, 0.5 g L^{-1} $MgSO_4 \cdot 7H_2O$, 0.02 g L^{-1} $FeSO_4 \cdot 7H_2O$, 0.02 g L^{-1} $MnSO_4$. The feed medium contained 500 g L^{-1} glucose (supplied by corn starch hydrolysate), 20 g L^{-1} corn steep liquor, 20 g L^{-1} $(NH_4)_2SO_4$. The feed medium was fed into the fermenter when the residual concentration of

glucose became below 20 g L^{-1}. The fed-batch fermentation was performed at 30 ^{o}C; pH was maintained at 7.0 by automatically adding 30% NH_4OH solution; the agitation speed was controlled at 220 rpm and the air flow rate was maintained at 500 L min^{-1}.

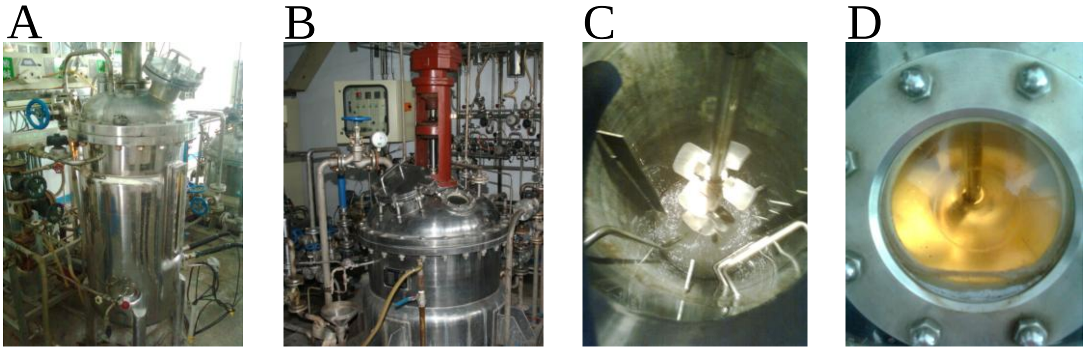

Figure S1. Pilot-scale L-arginine fed-batch fermentation. (**A**) The 100 L bioreactor for seed culture cultivation. (**B**) The appearance of the 1000 L bioreactor for L-arginine fed-batch fermentation. (**C**) The interior structure of the 1000 L bioreactor for L-arginine fed-batch fermentation. (**D**) The culture broth of L-arginine fermentation in the 1000 L bioreactor.

Table S1. Arginine batch fermentation of Cc0 and CcMB strains

**Strain**	**Arginine (g L^{-1})**	**Time (h)**	**Yield (g g^{-1})**	**DCW (g L^{-1})**
Cc0	14.2 ±0.83	80	0.147 ±0.009	17 ±0.56
CcMB	15.9 ±0.47	75	0.163 ±0.012	16.3 ±0.23

SDs based on three biologically independent experiments.

Table S2. Arginine batch fermentation of CcMB and Cc1 strains

**Strain**	**Arginine (g L^{-1})**	**Time (h)**	**Yield (g g^{-1})**	**DCW (g L^{-1})**
CcMB	15.1 ±0.53	72	0.161 ±0.012	16.1 ±0.38
Cc1	16.9 ±0.58	72	0.172 ±0.010	15.5 ±0.31

SDs based on three biologically independent experiments.

Table S3. Arginine batch fermentation of Cc1 and Cc1-2*pfk* strains

Strain	Specific PFK activities (U mg^{-1})	Arginine (g L^{-1})	Time (h)	Yield (g g^{-1})	DCW (g L^{-1})	NADPH level (μmol g^{-1} DCW)
Cc1	0.42±0.11	17.3±0.61	73	0.175±0.011	15.1±0.37	2.26±0.18
Cc1-2*pfk*	0.76±0.15	14.8±0.57	70	0.153±0.008	18.7±0.45	1.87±0.13

SDs based on three biologically independent experiments.

Table S4. Arginine batch fermentation of Cc1 and Cc2 strains

Strains	Specific PGI activities (U mg^{-1})	Arginine (g L^{-1})	Time (h)	Residual glucose (g L^{-1})	Yield (g g^{-1})	DCW (g L^{-1})	NADPH level (μmol g^{-1} DCW)
Cc1	1.53±0.17	16.7±0.31	72	3.3±1.2	0.181±0.010	15.6±0.46	2.31±0.15
Cc2-4000	0.48±0.11	13.5±0.28	84	32.5±2.3	0.198±0.008	9.8±0.32	3.69±0.27
Cc2-5000	0.54±0.06	16.4±0.25	84	19.6±1.8	0.201±0.011	11.4±0.22	3.51±0.21
Cc2-6500	0.87±0.08	18.8±0.36	84	8.0±3.1	0.197±0.012	13.2±0.41	3.17±0.19
Cc2-8500	1.06±0.15	18.3±0.42	76	5.7±2.3	0.186±0.010	14.8±0.32	2.73±0.25

SDs based on three biologically independent experiments.

Table S5. Arginine batch fermentation of Cc2-6500 and Cc2-G_{sod} strains

Strain	Arginine (g L^{-1})	Time (h)	Residual glucose (g L^{-1})	Yield (g g^{-1})	DCW (g L^{-1})
Cc2-6500	17.4±0.61	80	16.3±4.1	0.198±0.012	12.8±0.58
Cc2-G_{sod}	18.2±0.57	72	8.7±4.1	0.193±0.010	16.4±0.35

SDs based on three biologically independent experiments.

Table S6. Arginine batch fermentation of Cc2-G_{sod} and Cc3 strains

Strain	Arginine (g L^{-1})	Time (h)	Residual glucose (g L^{-1})	Yield (g g^{-1})	DCW (g L^{-1})
Cc2-G_{sod}	16.9±0.34	70	13.4±4.1	0.196±0.011	15.8±0.45
Cc3	19.0±0.46	70	2.3±1.4	0.194±0.013	17.9±0.61

SDs based on three biologically independent experiments.

Table S7. Arginine batch fermentation of Cc3 and Cc3-pyc_{G1A} strains

Strain	Specific PYC activities (U mg^{-1})	Arginine (g L^{-1})	Time (h)	Residual glucose (g L^{-1})	Yield (g g^{-1})	DCW (g L^{-1})
Cc3	0.33±0.12	16.9±0.34	65	13.4±5.3	0.193±0.008	16.4±0.65
Cc-pyc_{G1A}	0.71±0.23	18.7±0.46	65	2.2±1.2	0.189±0.011	18.2±0.47

SDs based on three biologically independent experiments.

Table S8. Arginine batch fermentation of Cc3-*pyc*$_{G1A}$ and Cc4 strains

Strain	Specific CS activities (U mg^{-1})	Arginine (g L^{-1})	Time (h)	Residual glucose (g L^{-1})	Yield (g g^{-1})	DCW (g L^{-1})
Cc-*pyc*$_{G1A}$	0.64±0.19	17.1±0.28	60	8.7±3.4	0.185±0.009	17.8±0.43
Cc4	1.05±0.21	18.4±0.35	60	4.8±2.8	0.195±0.012	18.7±0.56

SDs based on three biologically independent experiments.

Table S9. Arginine batch fermentation of Cc4 and Cc4-2*icd* strains

Strain	Specific ICD activities (U mg^{-1})	Arginine (g L^{-1})	Time (h)	Residual glucose (g L^{-1})	Yield (g g^{-1})	DCW (g L^{-1})	NADPH level (μmol g^{-1} DCW)
Cc4	0.89±0.16	18.1±0.28	60	7.6±2.8	0.198±0.013	18.4±0.52	3.26±0.27
Cc4-2*icd*	1.43±0.25	19.7±0.35	60	2.5±1.2	0.205±0.011	19.3±0.63	3.42±0.23

SDs based on three biologically independent experiments.

Table S10. Arginine batch fermentation of Cc4-2*icd* and Cc4-2*icd*-2*gdh* strains

Strain	Specific GDH activities (U mg^{-1})	Arginine (g L^{-1})	Time (h)	Residual glucose (g L^{-1})	Yield (g g^{-1})	DCW (g L^{-1})	Specific arginine yield (g g^{-1} DCW)
Cc4-2*icd*	1.87±0.39	20.3±0.37	60	4.6±2.1	0.208±0.012	19.8±0.37	1.02±0.09
Cc4-2*icd*-2*gdh*	3.16±0.54	21.1±0.35	60	8.5±1.2	0.226±0.014	18.7±0.32	1.15±0.08

SDs based on three biologically independent experiments.

Table S11. Arginine batch fermentation of Cc4-2*icd*-2*gdh* and Cc5 strains

Strains	Specific ODHC activities (U mg^{-1})	Arginine (g L^{-1})	Time (h)	Residual glucose (g L^{-1})	Yield (g g^{-1})	DCW (g L^{-1})	Specific arginine yield (g g^{-1} DCW)
Cc4-2*icd*-2*gdh*	1.28±0.24	21.4±0.26	60	6.7±1.2	0.228±0.011	18.6±0.54	1.15±0.07
Cc5-200	0.31±0.05	14.5±0.18	65	45.2±5.8	0.255±0.013	9.7±0.32	1.50±0.12
Cc5-500	0.42±0.08	21.7±0.35	65	20.8±2.5	0.254±0.015	14.4±0.33	1.51±0.11
Cc5-800	0.73±0.13	23.8±0.29	65	6.3±3.1	0.251±0.014	16.5±0.39	1.44±0.13
Cc2-1200	0.96±0.17	22.5±0.31	65	4.2±2.3	0.237±0.013	17.8±0.41	1.25±0.15

SDs based on three biologically independent experiments.

Table S12. Arginine batch fermentation of Cc5-800 and Cc5*lysC* strains

Strains	Specific AK activities (U mg^{-1})	Arginine (g L^{-1})	Time (h)	Residual glucose (g L^{-1})	Yield (g g^{-1})	DCW (g L^{-1})	Lysine (g L^{-1})	Isoleucine (g L^{-1})
Cc5-800	0.402±0.072	23.4±0.32	65	6.4±2.5	0.247±0.014	16.8±0.44	1.45±0.34	1.27±0.21
Cc5*lysC*-15	0.063±0.016	21.5±0.38	65	18.5±4.3	0.267±0.013	10.4±0.37	0.21±0.05	0.18±0.06
Cc5*lysC*-30	0.147±0.041	25.8±0.36	65	6.1±2.6	0.273±0.016	13.9±0.28	0.32±0.09	0.24±0.07
Cc5*lysC*-60	0.226±0.053	25.4±0.43	65	2.3±1.2	0.258±0.012	15.3±0.52	0.82±0.15	0.67±0.24
Cc5*lysC*-100	0.316±0.056	24.2±0.27	65	3.2±1.8	0.251±0.015	15.8±0.42	1.18±0.26	1.05±0.28

SDs based on three biologically independent experiments.

Table S13. Arginine batch fermentation of Cc5*lysC*-30 and Cc6 strains

Strain	Specific GK activities (U mg^{-1})	Arginine (g L^{-1})	Time (h)	Residual glucose (g L^{-1})	Yield (g g^{-1})	Proline (g L^{-1})
Cc5*lysC*-30	0.32±0.08	26.0±0.46	60	3.7±2.5	0.269±0.014	0.87±0.23
Cc6	0.03±0.01	27.3±0.35	60	5.2±2.1	0.284±0.017	Not detected

SDs based on three biologically independent experiments.

Table S14. The predicted cost of food-grade L-arginine production by fermentation of Cc6 strain

Item	Cost (RMB ton^{-1} L-arginine)
Material (medium and ammonia *etc.*)	16,000±1,500
Power (water, electricity and steam *etc.*)	15,000±1,000
Purification	5,000±500
Labor, equipment and sewage treatment *etc.*	8,000±500
Others	2,000±500
Total	46,000±4,000

The price of food-grade L-arginine produced by keratin hydrolysis method is 55,000-60,000 RMB ton^{-1} in China now.

Table S15. Strains used in this study

Strains	Relevant characteristics	Sources or references
***C. crenatum* strains**		
Cc0	Parent strain *C. crenatum* SYPA5-5	3,4
CcMB	Cc0+replacement of the natural *argB* gene encoding N-acetylglutamate kinase by mutated *argB*$_{M3}$ gene	This study
Cc1	CcMB+replacement of the natural promoters of the *argCJBDFR* and *argGH* operons by the *eftu* promoter	This study
Cc1-2*pfk*	Cc1+ additional copy of the *pfk* gene encoding phosphofructokinase	This study
Cc2	Cc1+replacement of the natural RBS of *pgi* gene encoding phosphoglucoisomerase by weaker RBS	This study
Cc2-4000	Cc1+replacement of the natural RBS of *pgi* gene by 4000 au of activity RBS	This study
Cc2-5000	Cc1+replacement of the natural RBS of *pgi* gene by 5000 au of activity RBS	This study
Cc2-6500	Cc1+replacement of the natural RBS of *pgi* gene by 6500 au of activity RBS	This study
Cc2-8500	Cc1+replacement of the natural RBS of *pgi* gene by 8500 au of activity RBS	This study
Cc2-G$_{sod}$	Cc2-6500+replacement of the natural promoter of the *ptsG* gene encoding glucose-specific EIIABC^{Glc} component by the *sod* promoter	This study
Cc3	Cc2-G$_{sod}$+replacement of the natural promoters of the *iolT1* gene encoding inositol permeases and *ppgk* gene encoding glucose kinases by the *sod* promoter	This study
Cc3-*pyc*$_{G1A}$	Cc3+replacement of the start codon GTG by ATG in the *pyc* gene encoding pyruvate carboxylase	This study
Cc4	Cc3-*pyc*$_{G1A}$+additional copy of the *gltA* gene encoding citrate synthase	This study
Cc4-2*icd*	Cc4+additional copy of the *icd* gene encoding isocitrate dehydrogenase	This study
Cc4-2*icd*-2*gdh*	Cc4-2*icd*+additional copy of the *gdh* gene encoding glutamate dehydrogenase	This study
Cc5	Cc4-2*icd*-2*gdh*+replacement of the natural RBS of *odhA* gene encoding α-ketoglutarate dehydrogenase by weaker RBS	This study
Cc5-200	Cc4-2*icd*-2*gdh*+replacement of the natural RBS of *odhA* gene by 200 au of activity	This study

	RBS	
Cc5-500	Cc4-2*icd*-2*gdh*+replacement of the natural RBS of *odhA* gene by 500 au of activity RBS	This study
Cc5-800	Cc4-2*icd*-2*gdh*+replacement of the natural RBS of *odhA* gene by 800 au of activity RBS	This study
Cc5-1200	Cc4-2*icd*-2*gdh*+replacement of the natural RBS of *odhA* gene by 1200 au of activity RBS	This study
Cc5*lysC*	Cc5-800+replacement of the natural RBS of *lysC* gene encoding aspartokinase by weaker RBS	This study
Cc5*lysC*-15	Cc5-800+replacement of the natural RBS of *lysC* gene by 15 au of activity RBS	This study
Cc5*lysC*-30	Cc5-800+replacement of the natural RBS of *lysC* gene by 30 au of activity RBS	This study
Cc5*lysC*-60	Cc5-800+replacement of the natural RBS of *lysC* gene by 60 au of activity RBS	This study
Cc5*lysC*-100	Cc5-800+replacement of the natural RBS of *lysC* gene by 100 au of activity RBS	This study
Cc6	Cc5*lysC*-30+deletion of *proB* gene encoding glutamate kinase	This study
***E. coli* strain**		
JM109	Wild type *E. coli*	TaKaRa

Table S16. Plasmids used in this study

**Plasmids**	**Relevant characteristics**	**Sources or references**
pK18	pK18*mobsacB* , Kan^r, vector for allelic exchange in *C. glutamicum*	5
pK18-*argB*$_{M3}$	pK18 derivative for chromosomal replacement of natural *argB* gene by mutated *argB*$_{M3}$ gene	This study
pK18-P$_{eftu}$*argCJBDFR*	pK18 derivative for chromosomal replacement of *argCJBDFR* operon promoter by *eftu* promoter	This study
pK18-P$_{eftu}$*argGH*	pK18 derivative for chromosomal replacement of *argGH* operon promoter by *eftu* promoter	This study
pK18-2*pfk*	pK18 derivative for implementation of an additional gene copy of *pfk*	This study
pK18-rbs*pgi*	pK18 derivative for chromosomal replacement of natural RBS of *pgi* gene by weaker RBS	This study
pK18-4000rbs*pgi*	pK18 derivative for chromosomal replacement of natural RBS of *pgi* gene by 4000 au of activity RBS	This study
pK18-5000rbs*pgi*	pK18 derivative for chromosomal replacement of natural RBS of *pgi* gene by 5000 au of activity RBS	This study
pK18-6500rbs*pgi*	pK18 derivative for chromosomal replacement of natural RBS of *pgi* gene by 6500 au of activity RBS	This study
pK18-8500rbs*pgi*	pK18 derivative for chromosomal replacement of natural RBS of *pgi* gene by 8500 au of activity RBS	This study
pK18-P$_{sod}$*ptsG*	pK18 derivative for chromosomal replacement of *ptsG* gene promoter by *sod* promoter	This study
pK18-P$_{sod}$*iolT1*	pK18 derivative for chromosomal replacement of *iolT1* gene promoter by *sod* promoter	This study
pK18-P$_{sod}$*ppgk*	pK18 derivative for chromosomal replacement of *ppgk* gene promoter by *sod* promoter	This study
pK18-ATG*pyc*	pK18 derivative for chromosomal replacement of the natural start codon GTG of *pyc* gene by ATG	This study
pK18-2*gltA*	pK18 derivative for implementation of an additional gene copy of *gltA*	This study
pK18-2*icd*	pK18 derivative for implementation of an additional gene copy of *icd*	This study
pK18-2*gdh*	pK18 derivative for implementation of an additional gene copy of *gdh*	This study
pK18-rbs*odhA*	pK18 derivative for chromosomal replacement of natural RBS of *odhA* gene by weaker RBS	This study
pK18-200rbs*odhA*	pK18 derivative for chromosomal replacement of natural RBS of *odhA* gene by 200 au of activity RBS	This study

pK18-500rbs*odhA*	pK18 derivative for chromosomal replacement of natural RBS of *odhA* gene by 500 au of activity RBS	This study
pK18-800rbs*odhA*	pK18 derivative for chromosomal replacement of natural RBS of *odhA* gene by 800 au of activity RBS	This study
pK18-1200rbs*odhA*	pK18 derivative for chromosomal replacement of natural RBS of *odhA* gene by 1200 au of activity RBS	This study
pK18-rbs*lysC*	pK18 derivative for chromosomal replacement of natural RBS of *lysC* gene by weak RBS	This study
pK18-15rbs*lysC*	pK18 derivative for chromosomal replacement of natural RBS of *lysC* gene by 15 au of activity RBS	This study
pK18-30rbs*lysC*	pK18 derivative for chromosomal replacement of natural RBS of *lysC* gene by 30 au of activity RBS	This study
pK18-60rbs*lysC*	pK18 derivative for chromosomal replacement of natural RBS of *lysC* gene by 60 au of activity RBS	This study
pK18-100rbs*lysC*	pK18 derivative for chromosomal replacement of natural RBS of *lysC* gene by 100 au of activity RBS	This study
pK18-Δ*proB*	pK18 derivative for in-frame deletion of *proB* gene	This study

Table S17. Primers used in this study

Name	DNA Sequence (5'-3')	Restriction site
argB-1	CGCGAATTCATGAATGACTTGATCAAAG	*EcoR*I
argB-2	CGCGTCGACTTACAGTTCCCCATCCTTG	*Sal*I
argC-1	CCGGAATTCAGGAATCTTCCGCAGTTGAA	*EcoR*I
argC-2	CATTCGCAGGGTAACGGCCAATGCATAATTTTTTGATTCA	
Peftu-1	TGGCCGTTACCCTGCGAATG	
Peftu-2	TGTATGTCCTCCTGGACTTC	
argC-3	GAAGTCCAGGAGGACATACAGAATAATTTGCATGATCATG	
argC-4	CCCAAGCTTTTTTGCAGACGAAAGTCAGC	*Hind*III
argG-1	CCGGAATTCCGCATGCTTGATGAACTGCTG	*EcoR*I
argG-2	CATTCGCAGGGTAACGGCCAAGGATGTTTAAAGAAAATTA	
argG-3	GAAGTCCAGGAGGACATACATGCTGTCCACCACGGCTGGC	
argG-4	CCCAAGCTTACGAACCTGGTCGTTGCCCT	*Hind*III
pfk-1	CCGGAATTCCAAACCTGCTGCGACGACGG	*EcoR*I
pfk-2	ATTTCACTTACTTGCACCTT	
pfk-3	AAGGTGCAAGTAAGTGAAATTGGCAGTGCTGACCTGGGAT	
pfk-4	CGCGGATCCCAATGGATCTATGCCACTTG	*BamH*I
pgi-1	CCGGAATTCTCTCGATCCCTTCTCCGGCA	*EcoR*I
pgi-2	AAATAACATCTTCAGGTTAGCTT	
pgi-3	AAGCTAACCTGAAGATGTTATTT-Sequence of Synthetic RBS-ATGGCGGACATTTCGACCACCC	

Sequence of Synthetic RBS:

4000 au: ACAACAAAATTAAACACCACGAAAGGAGTATAGGC
5000 au: TAATCCACTACGATAAAGGAAATACCAATA
6500 au: GGCGTTTCACTACACAAGGAAGGATATTAACGTAC
8500 au: TTTCAATAAGAATCAGAAGATATAGGACAGATTT

pgi-4	CCCAAGCTTTGAGCGCAGCGCAGTAGCGA	*Hind*III
Psod-1	CTACTCAGCTGCCGATTATTCCGGG	
Psod-2	GGGTAAAAATCCTTTCGTAG	
ptsG-1	CGCGAATTCGAGGGGTGGTGCGGGCATAA	*Eco*RI
ptsG-2	CCCGGAATAATCGGCAGCTGAGTAGTCTCCCGCGGGCAAGAGACT	
ptsG-3	CTACGAAAGGATTTTTACCCCAGACCCTACGTTTAGAAAG	
ptsG-4	CCCAAGCTTAACTCGAAGGCGTAGTCAAT	*Hind*III
iolT1-1	CCGGAATTCTCTTTAAGCAGTGAATGAGG	*Eco*RI
iolT1-2	CCCGGAATAATCGGCAGCTGAGTAGTTACTTGGAGGTGCAGGGTC	
iolT1-3	CTACGAAAGGATTTTTACCCGGGCACGACAAACTTAGGAG	
iolT1-4	CCCAAGCTTATTACTGCAAAAGATGGAGC	*Hind*III
ppgk-1	CCGGAATTCCAGGTAACTCCGCTTATCGA	*Eco*RI
ppgk-2	CCCGGAATAATCGGCAGCTGAGTAGTCGGCTTTTGGCAGGCAATT	
ppgk-3	CTACGAAAGGATTTTTACCCAGTAATTTGTACACTTGGGC	
ppgk-4	CCCAAGCTTGAGAACGGTGATCTCTCGGC	*Hind*III
pyc-1	CCGGAATTCTGAGTCTCAGATTTTAAGAA	*Eco*RI
pyc-2	TAGAGTAATTGTTCCTTTCA	
pyc-3	TGAAAGGAACAATTACTCTAATGTCGACTCACACATCTTC	
pyc-4	CCCAAGCTTTTATCACCGGTGAGATCAAG	*Hind*III
gltA-1	CCGGAATTCAGCTACTCGGCACCCATCCT	*Eco*RI
gltA-2	AGTTTGTGATGCACACGACATGTACATGCTCCTTGAAAAT	
gltA-3	ATTTTCAAGGAGCATGTACATGTCGTGTGCATCACAAACT	
gltA-4	CCCAAGCTTTCTGTGGCTTTTCCATGTGC	*Hind*III
icd-1	CCGGAATTCCCACCATATGGAGGAAGACG	*Eco*RI
icd-2	GAGTTCTTCGGTGGCGGCGGATGCAGGAAGAACTCGAGAA	
icd-3	TTCTCGAGTTCTTCCTGCATCCGCCGCCACCGAAGAACTC	
icd-4	CTAGTCTAGACACGGCGGTAGTGATGAGAA	*Xba*I
gdh-1	CCGGAATTCTTTAATTCTTTGTGGTCATA	*Eco*RI
gdh-2	TTATGGCAGCGTCGCACAGATGGATCGGATAATTGACCTG	
gdh-3	CAGGTCAATTATCCGATCCATCTGTGCGACGCTGCCATAA	

gdh-4	CCCAAGCTTCACAGCTGCATTAACCCATG	*Hind*III
odhA-1	CCGGAATTCCGGACAGCGCCAACTGATCG	*Eco*RI
odhA-2	ACGTGTCAGGCCATTAAATG	
odhA-3	CATTTAATGGCCTGACACGT-Sequence of Synthetic RBS-ATGCTACAACTGGGGCTTAGGC<br>**Sequence of synthetic RBS**<br>200 au: ACGTAGAGTATCTATCAGCCCGTCAAGGA<br>500 au: ATAGTAAATACAGCCTAACTAGGCCAGAA<br>800 au: ATTTATATTCACAAATAGTTAAACGATAGACGTA<br>1200 au: CAACTTCTTAATTTAATACTAAAAATAATAAT	
odhA-4	CGCGGATCCTGAAAATACCCCTGATTGGG	*BamH*I
lysC-1	CCGGAATTCCCCAGAAGATTTCAGTTCTG	*Eco*RI
lysC-2	CGTGCTGACAGTTACCCGCT	
lysC-3	AGCGGGTAACTGTCAGCACG-sequence of synthetic RBS-GTGGCCCTGGTCGTACAGA<br>**Sequence of synthetic RBS**<br>15 au: CGCTTCCAGTTTTTATAACCGAAAGAAAGAA<br>30 au: AATTAACTAGAATAAAAATTTAGTTAAGGACATAG<br>60 au: CATTAGTTCTAGCCTCTAAGATAAGAGAACAA<br>100 au: CTTTCGGTCACAGGACTAACCATCAGGTTCAACA	
lysC-4	CCCAAGCTTCACACATCAGCGTTCAGAGC	*Hind*III
proB-1	CCGGAATTCATGGCGCCGGTGACAGGGCT	*Eco*RI
proB-2	GGCGTCTTCCAGTGCTGGGCGGTGCTCAATCCAAGCGGGG	
proB-3	CCCCGCTTGGATTGAGCACCGCCCAGCACTGGAAGACGCC	
proB-4	CCCAAGCTTTTACGCGCGACTGGCGTAGT	*Hind*III

Introduced restriction sites are underlined.

Table S18. Primers used for the RT-PCR experiments[6]

Name	DNA Sequence (5'-3')
argC-F	GCCACAAACCCAAGCAGTTC
argC-R	CGATTGCGGAGGTAACAAGG
argJ-F	TGTTTGACCACTGACGCATC
argJ-R	GCATCGTTGAGTTCATCC
argB-F	GATGCTAAACCGTGTGGG
argB-R	ACCGACCTGACCAAAGAG
argD-F	ACAAGCGTGAAGCGTTCCTG
argD-R	ATAGCAGCCACATCCGTTGG
argF-F	GTCATCACCGATACCTGG
argF-R	ACGCTGGTCCATCAATCACG

argG-F	GATGAGTTCGCTGAGGAG
argG-R	AACCTGGTCGTTGCCCTTAC
argH-F	GCAGCCGATAACTCCATTG
argH-R	GACCAGGAATCAGACAAGG
pfk-F	AGTGGCTGTCTGATAACGGT
pfk-R	CCATGACCTCCACAATCATC
pgi-F	ACGGACAACATGTTCGGCTT
pgi-R	GCAGAGCCATCAAGATTGGA
ptsG-F	TGGATGAGCAGCCTGATACT
ptsG-R	GCCAAAGACTGTGACGGTAT
iolT1-F	GTTGGTCAGCTCGCAGCTTT
iolT1-R	CTCATCAATGCGTCCTCGCT
ppgk-F	CCTTGGTACAGGTATTGGATC
ppgk-R	GTTTCTCGTATTCGCTCAGCA
pyc-F	CACTTTGACTCCATGCTGGTG
pyc-R	CGATGCGCTTGGAAGTGAAG
gltA-F	CCTACTACCAGGATCAGCTGAAC
gltA-R	GTATGGCTCGGTTGGGTAAC
icd-F	ATACCGTCACCATTAAGCAC
icd-R	CTTCATCATGGTGGCCTTC
gdh-F	CTGCACTTGGACCATACAAG
gdh-R	CGCATGATTTCCAGATCG
odhA-F	GATTCCTTCTGGGACGAGATC
odhA-R	CATGAAAGTGGGTTGGTGTC
lysC-F	GACATGGTTCTGCAGAACGTC
lysC-R	CATGAACTCTGCGGTAAC
16S rRNA-F	GCCCAGGTAAGGTTCTTC
16S rRNA-R	GGTGTAGCGGTGAAATGC

References

1 Becker, J., Zelder, O., Häfner, S., Schröder, H. & Wittmann, C. From zero to hero—Design-based systems metabolic engineering of *Corynebacterium glutamicum* for l-lysine production. *Metab. Eng.* **13**, 159-168 (2011).

2 Park, S. H. *et al.* Metabolic engineering of *Corynebacterium glutamicum* for L-arginine production. *Nat. Commun.* **5**, 1-9 (2014).

3 Xu, M. *et al.* Site-directed mutagenesis and feedback-resistant N-acetyl-L-glutamate kinase (NAGK) increase *Corynebacterium crenatum* L-arginine production. *Amino Acids* **43**, 255-266 (2012).

4 Xu, H. *et al.* A two-stage oxygen supply strategy for enhanced l-arginine production by *Corynebacterium crenatum* based on metabolic fluxes analysis. *Biochem.Eng. J.* **43**, 41-51 (2009).
5 Schäfer, A. *et al.* Small mobilizable multi-purpose cloning vectors derived from the Escherichia coli plasmids pK18 and pK19: selection of defined deletions in the chromosome of *Corynebacterium glutamicum*. *Gene* **145**, 69-73 (1994).
6 Xu, M., Rao, Z., Dou, W. & Xu, Z. The role of ARGR repressor regulation on L-arginine production in *Corynebacterium crenatum*. *Appl. Biochem. Biotechnol.* **170**, 587-597 (2013).
